# Novel fabrication of SiO_2_/Ag nanocomposite by gamma irradiated *Fusarium oxysporum* to combat *Ralstonia solanacearum*

**DOI:** 10.1186/s13568-022-01372-3

**Published:** 2022-02-28

**Authors:** Amira G. Zaki, Yasmeen A. Hasanien, Gharieb S. El-Sayyad

**Affiliations:** 1 Plant Research Department, Nuclear Research Center (NRC), Egyptian Atomic Energy Authority (EAEA), Cairo, Egypt; 2 Drug Radiation Research Department, National Center for Radiation Research and Technology (NCRRT), Egyptian Atomic Energy Authority (EAEA), Cairo, Egypt

**Keywords:** Bacterial wilt, *Ralstonia solanacearum*, Silica/silver nanocomposite, Solid-state fermentation, *Fusarium oxysporum*, Gamma irradiation

## Abstract

The bacterial wilt is a global destructive plant disease that initiated by the phytopathogenic *Ralstonia solanacearum*. This study display a novel biofabrication of silica/silver nanocomposite using *Fusarium oxysporum*-fermented rice husk (RH) under solid state fermentation (SSF). The biofabricated nanocomposite was characterized by XRD, UV–Vis. spectroscopy, DLS, SEM, EDX elemental mapping, and TEM analyses as well as investigated for anti-*R. solanacearum* activity. Response surface methodology was also processed for optimizing the biofabrication process and improving the anti-bacterial activity of the fabricated nanocomposite. Maximum suppression zone of 29.5 mm against *R. solanacearum* was reached at optimum RH content of 6.0 g, AgNO_3_ concentration of 2.50 mM, reaction pH of 6.3, and reaction time of 2 days. The anti-*R. solanacearum* activity of the fabricated nanocomposite was further improved by exposing the *F. oxysporum* strain to a gamma irradiation dose of 200 Gy. In conclusion, RH recycling under SSF by *F. oxysporum* could provide an innovative, facile, non-expensive, and green approach for fabricating SiO_2_/Ag nanocomposite that could be applied efficiently as an eco-friendly antibacterial agent to combat *R. solanacearum* in agricultural applications. Moreover, the developed method could serve as a significant platform for the designing of new nanostructures for broad applications.

**Supplementary Information:**

The online version contains supplementary material available at 10.1186/s13568-022-01372-3.

## Key points

- Biofabricated SiO_2_/Ag nanocomposite by gamma irradiated-*F. oxysporum* under SSF.
- Statistical optimization and complete characterization of SiO_2_/Ag nanocomposite.
- Application in agriculture for combating the wilt causing *R. solanacearum.*

**Supplementary Information:**

The online version contains supplementary material available at 10.1186/s13568-022-01372-3.

## Introduction

The *Ralstonia solanacearum* wilting of *Solanaceae* has become one of the most common and catastrophic agricultural diseases, affecting a significant number of important economical agricultural crops such as tomatoes, eggplants, potatoes, tobacco (Chen et al. 2016; Cheng et al. 2020) etc. The pathogen *R. solanacearum* invades the injured lateral roots, and diffuses along the host plant via bacterial colonization of the stem and crown, causing symptoms of wilt (Chen et al. 2016). Reports demonstrated that main virulence factors, such as adhesin-like proteins, plant cell wall-degrading enzymes, polysaccharides, phyto-hormones, and reactive oxygen species (ROS), participate significantly to the bacterial wilting pathogenesis (Chen et al. 2016, 2019).

Currently, the protection of crops from *R. solanacearum* includes application of pesticides and synthetic fertilizers. Over-application of these synthetic compounds would damage the environment, public hygiene and create pesticide resistance (Sun et al. 2012). Replication of *R. solanacearum*-resistant plant lines is investigated as one of the most prospective control strategies. Several genes regulating bacterial wilt resistance in tomatoes have been recognized previously (Thoquet et al. 1996). *RRS1-R*, a recessive gene in the *Arabidopsis thaliana* plant allows a broad spectrum resistance to *R. solanacearum* was identified by Deslandes et al. (2002). Moreover, a crop rotation protocol has been applied extensively to reduce the incidence of bacterial wilt. Recently, investigation of innovative approaches such as nanotechnology represents a prospective alternative to pesticides for controlling bacterial wilting (Attia et al. 2019, 2021; El-Batal et al. 2020b).

The design and managed preparation of nanocomposites has attained significant investigation over the past few years and has become a vital zone of research in various field as material science, medicine, engineering, chemistry (Abd Elkodous et al. 2019; Wu et al. 2016). Nano-Ag composites via immobilization of nano-Ag on chemical materials, agro-industrial remains, or other matrices have been employed for the fabrication of new materials to be applied in wound dressings, paints, textile industrial, domestic cleaning solutions, water treatment, electrical and electronics, refrigerators, washing machines, and cosmetics (Thamilselvi and Radha 2017; and references therein).

Silica/silver (SiO_2_/Ag) structures consisting of silver nanoparticles (NPs) immobilized on silica nanospheres are important among the different types of nanocomposite structured materials due to their specific properties and possible applications especially in biological and medicinal field (Qin et al. 2017; Tudose et al. 2017), and optical devices (Wu et al. 2016). Different synthesis protocols for SiO_2_/Ag nanocomposite were tried including chemical reduction process, layer by layer (LBL), one-pot synthesis, sol–gel technique, template, micro-emulsion technique, thermal deposition (Liu et al. 2011; Tian et al. 2014; Wu et al. 2016) etc. The synthesis, catalytic properties, and antimicrobial activity of SiO_2_/Ag nanocomposite was reported (Han et al. 2014; Wu et al. 2016; Qin et al. 2017). Since the preparation method for the SiO_2_/Ag nanocomposite includes a complex and time- and effort-consuming several steps, there is necessity to develop a facile, green, cost effective, and easily manipulated method for the SiO_2_/Ag nanocomposite fabrication with a size controlled nano-scaled Ag layer (Wu et al. 2016).

Solid-state fermentation (SSF) is a three-phase complex manner including solid, liquid, and gaseous states, which helps microbial cultivation for bioprocesses and product improvement. Across the last two decades, SSF has achieved important consideration for developing manufacturing bioprocesses, mainly because of the lower energy demands for more precious output yields and less wastewater generation with a lesser hazard of bacterial infection. In addition, it is eco-friendly, as it frequently uses solid wastes from agro-industrial systems as the substrate (Thomas et al. 2013). Rice husk (RH) is a widely used agricultural waste, and its annual world yield is around 120 million tons per year. It is estimated to contain about 20% SiO_2_, making it a prospective renewable source for obtaining SiO_2_ (Cui et al. 2016). Herein, *Fusarium oxysporum*-fermented rice (FRH) husk under SSF was employed for the first time for biosynthesizing amorphous nano-SiO_2_ spheres as well as for reducing Ag ions in AgNO_3_ solution into Ag NPs over the surface of nano-SiO_2_ spheres to bio-fabricate SiO_2_/Ag nanocomposite. The process conditions including RH weight, AgNO_3_ concentration, reaction pH, and reaction time were statistically adjusted using response surface methodology for efficient biofabrication of SiO_2_/Ag nanocomposite with a maximum activity against the plant pathogen *R.* s*olanacearum.* Moreover, different gamma irradiation doses were screened for improving the biosynthesis proficiency by *F. oxysporum* strain.

## Materials and methods

### Microorganism

The fungal strain used in the biosynthesis of the nanocomposite was *F. oxysporum* (AUMC 10544, purchased from the Assuit University Mycological Center for culture collection, http://www.aun.edu.eg/aumc.htm, Assuit, Egypt). The strain was cultivated on potato dextrose agar (PDA) slants composed of (g L^−1^): potato infusion 200, dextrose 20, and agar 20 at 25 °C for 5 days. After cultivation, slants were flooded with a sterile saline, vigorously vortexed, then gone through a serial diluting and counting using a heamocytometer for attaining 1 × 10^8^ spores mL^−1^.

### Silica bioleaching under solid‑state fermentation

Rice husk (RH) was obtained from a rice milling plant at Zagazig, El-Sharqia, Egypt. Biosynthesis of nano-SiO_2_ from a fermented RH is processed here under solid state fermentation (SSF) through a modified two-step method of that reported by Bansal et al. (2006). Primarily, SSF was managed as following: weighted samples of 10 g of RH were separately transferred to 250 mL flasks then a solution of Czapex’s mineral salt that composed of NaNO_3_, 0.3; K_2_HPO_4_, 0.1; KCl, 0.05; MgSO_4_·7H_2_O, 0.05; FeSO_4_·7H_2_O, 0.001 (g%) was added for moistening the waste at a level of 70%, w/w. Then, flasks underwent autoclaving for 15 min at 121 °C. After cooling, flasks were inoculated by 1 mL of the adjusted fungal spore suspension followed by a gentle shaking of the flask contents. Flasks were then incubated at 25 °C for 4 days. The next step was for SiO_2_ leaching from the *F. oxysporum*-fermented RH. Accordingly, 100 mL of sterile distilled water were added to the fermented RH and the biotransformation process of the RH silica by *F. oxysporum* into nano-SiO_2_ was run at room temperature for 24 h in a 200 rpm shaker. The fermented waste was then separated from the aqueous component by filtration through Whatman no.1 filter paper (El-Gomhouria Company, Cairo, Egypt) and the resultant filtrate was centrifuged for 20 min at 10,000 rpm to remove the fermented waste impurities and then the collected pellet underwent sequential washing steps by a sterile distilled water and centrifugation. Finally it was left to dry at 65^o^ C-adjusted oven for 24 h.

### Fabrication of SiO_2_/Ag nanocomposite

Synthesizing and subsequent immobilization of nano-Ag on nano-SiO_2_ spheres for fabricating SiO_2_/Ag nanocomposite was processed based on the reported studies (Al-Askar et al. 2013; Cui et al. 2016; Lieu et al. 2018) with modifications. As the processed SSF culture is rich by *F. oxysporum* metabolites and the fermented RH extract, it would provide efficient reduction of silver nitrate and generating nano-Ag. Briefly, a SSF was processed again as described above and 100 mL of sterile distilled water were added to the fermented RH and left at room temperature for 24 h to generate nano-SiO_2_ spheres. After that, 100 mL of AgNO_3_ solution was supplemented followed by raising the pH using Na OH (1 N, w/v). The reduction process was run in dark at room temperature at 200 rpm shaker. Then, the flask contents underwent filtration through Whatman no.1 filter paper and resultant filtrate was centrifuged for 20 min at 10,000 rpm to remove the fermented waste impurities and then the collected pellet underwent several steps of washing by sterile distilled water and centrifugation. Finally it was left to dry at 65 °C-adjusted oven for 24 h.

### Biosynthesis of nano-Ag by *F. oxysporum*

In order to compare between the efficiency of Ag NPs alone and their activity after being immobilized on nano-SiO_2_ spheres in the biofabricated nanocomposite, nano-Ag were synthesized alone by *F. oxysporum* under submerged fermentation according to the method described by Al-Askar et al. (2013). Briefly, *F. oxysporum* was cultivated in potato-dextrose broth (PDP) for 1 week statically at 25 °C. Then, 10 mL *F. oxysporum* culture filtrate were transferred to a 250 mL Erlenmeyer flask containing 100 mL of 1 mM AgNO_3_ solution and were left in the dark on 200 rpm shaker at room temperature for 3 days. After that, the flask content was centrifuged at 10,000 rpm for 20 min. The collected pellet underwent subsequent steps of washing with sterile distilled water and centrifugation and then was left to dry at 65 °C-adjusted oven for 24 h.

### Characterization of the biosynthesized nano-SiO_2_ and SiO_2_/Ag nanocomposites

The stoichiometry of the synthesized nano-samples was examined using energy dispersive X-ray spectra (EDX), JEOL JSM-5600 LV, Japan. The crystal structure of the samples was investigated using X-ray diffraction (XRD) (Shimadzu XRD-6000, Japan). XRD patterns were obtained in a range of 2θ from 17° to 90° at room temperature. Cu Kα was used as the radiation source of wavelength λ = 0.15408 nm, with a scan rate of 0.8°/min, an operating voltage of 50 kV, and a current of 40 mA. Information about the shape and grain size of the sample particles were obtained using scanning electron microscopy (SEM) (JEOL JSM-5600 LV, Tokyo, Japan). The shape and size of the synthesized samples were obtained by transmission electron microscopy (TEM, JOEL JEM-1400, Tokyo, Japan) at 80 kV accelerating voltage. Finally, particle size distribution, the hydrodynamic radius, and polydispersity index (PDI) of the synthesized samples was determined by dynamic light scattering (DLS; Malvern Panalytical, Malvern, UK).

### Statistical optimization of SiO_2_/Ag nanocomposite biofabrication by response surface methodology

Response Surface Methodology (RSM) using Box Behnken design (BBD) was applied for detecting the optimum levels of the most effective factors in SiO_2_/Ag nanocomposite biofabrication under SSF by *F. oxysporum* resulting in maximum anti-*R. solanacearum* activity. In addition, it would be possible to investigate the relationships between the selected factors. The four selected critical factors were RH concentration, silver nitrate concentration, reaction pH value, and reaction time. Through BBD, each factor was investigated at different three coded levels (− 1, 0, + 1). Each design run was duplicated and the mean values of the estimated zone of inhibition (ZoI, mm) were statistically analyzed.

Thus, for a four-factor design, a total of 27 experimental trials were performed (Table 1) and the ZoI values were fitted to the following second order polynomial equation:

$$Y= {\beta }_{O}+\sum {\beta }_{i}{X}_{i}+\sum {\beta }_{ii}{X}_{i}^{2}+\sum {\beta }_{ij}{X}_{i}{X}_{j}+e$$

where Y is ZoI, mm; β_o_ a constant; β_*i*_, β_*ii*_, and β_*ij*_ are the linear, squared, and cross coefficient; X_*i*_ and X_*j*_ represents the investigated terms, and e refers to the residual term.

**Table 1:** Box Behnken design matrix with non-coded values of the studied parameters and the obtained measures of zone of inhibition (ZoI, mm)

Run	A: RH Conc.	B: AgNO_3_ Conc.	C: pH	D: Reaction time	Actual ZoI (mm)	FITS values
1	5	1	6.5	2	20.2 ± 1.44^d,e,f,g^	20.0
2	15	1	6.5	2	18.9 ± 0.55^g,h,i^	19.9
3	5	3	6.5	2	30.1* ± 1.25^a^	28.6
4	15	3	6.5	2	12.4 ± 1.50^l^	12.1
5	10	2	5	1	15.9 ± 0.55^j,k^	15.5
6	10	2	8	1	12.1 ± 1.25^l,m^	11.7
7	10	2	5	3	20.5 ± 1.00^d,e,f,g^	20.4
8	10	2	8	3	15.2 ± 1.31^k^	15.1
9	5	2	6.5	1	18.8 ± 1.60^g,h,i^	19.4
10	15	2	6.5	1	10.3 ± 1.52^m^	10.1
11	5	2	6.5	3	21.7 ± 0.26^d,e^	22.5
12	15	2	6.5	3	15.4 ± 0.52^k^	15.3
13	10	1	5	2	22.1 ± 1.44^c,d^	22.2
14	10	3	5	2	19.8 ± 0.28^e,f,g,h^	20.4
15	10	1	8	2	15.4 ± 1.50^k^	15.4
16	10	3	8	2	17.6 ± 0.40^i,j^	18.1
17	5	2	5	2	23.8 ± 1.60^c^	23.8
18	15	2	5	2	17.9 ± 0.55^h,i^	17.5
19	5	2	8	2	21.1 ± 1.25^d,e,f^	21.2
20	15	2	8	2	11.2 ± 1.31^l,m^	10.9
21	10	1	6.5	1	15.1 ± 1.44^k^	14.8
22	10	3	6.5	1	15.5 ± 0.55^k^	16.0
23	10	1	6.5	3	20.5 ± 1.00^d,e,f,g^	19.7
24	10	3	6.5	3	19.4 ± 0.50^f,g,h,i^	19.4
25	10	2	6.5	2	28.0 ± 1.00^b^	27.3
26	10	2	6.5	2	27.0 ± 1.25^b^	27.3
27	10	2	6.5	2	27.0 ± 1.00^b^	27.3

*RH* rice husk, *Conc*. concentration

FITS values are the values predicted by the model

Data for ZoI (zone of inhibition) are shown as the mean ± SD of triplicate measurements from independent experiments

a–m means with different superscripts in the same column are considered statistically different (LSD test, *P* ≤ 0.05)

*refers to the maximum ZoI (mm) obtained at run 3

The goodness of the constructed model was evaluated by the Analysis of Variance (ANOVA), model coefficient of determination (R^2^) using Minitab version 18.0 software (free trial version). Finally, the model obtains were tested through a validation experiment to authenticate the model accuracy.

The antibacterial assay of the biofabricated SiO_2_/Ag nanocomposite at each experimental trial of BBD was performed against the *R. solanacearum* EMCC 1274 strain obtained from the Microbiological Resources Centre (MIRCEN) at the Faculty of Agriculture, Ain Shams University, Cairo, Egypt. A well diffusion protocol was applied. Accordingly, Petri-dishes containing casamino acid-peptone-glucose (CPG) medium of the following composition (g L^−1^): casamino acid, 1; peptone, 10; glucose, 5; and agar 20 were surface inoculated with 0.1 mL bacterial suspension (10^5^ cell mL^−1^) were prepared. Then, SiO_2_/Ag nanocomposite suspension (50 µL; 1 mg mL^−1^) was applied to agar wells (8 mm, diameter). The biosynthesized nano-SiO_2_ only as well as the biosynthesized nano-Ag only were also applied to the agar wells as control. Plates were incubated at 7 °C overnight, then at 30 °C for 24–48 h and checked for zone of inhibition (ZoI, mm).

### Investigating the gamma irradiation effect

In this experiment, spore suspension of *F. oxysprum* were prepared as described above, distributed in paraffin sealed vials and undergone gamma irradiation at doses of 200, 400, 600, 800 Gy using ^60^Co Gamma chamber, MC20, Russia at the Nuclear Research Center (NRC), Egyptian Atomic Energy Authority (EAEA), Cairo, Egypt. The average dose rate was 478.15 Gy h^−1^ at the time of the experiment. After irradiation, the irradiated suspensions was maintained for 24 h at 7 °C to prevent the occurrence of photo–reactivation. Then, the irradiated suspension at each irradiation dose was separately solid-state cultivated and the biofabrication of SiO_2_/Ag was processed at the optimum conditions attained from RSM. Then, the anti-*R. solanacearum* activity was evaluated at each irradiation dose in triplicates to investigate the enhancement irradiation dose resulted in an enhanced antibacterial activity. Moreover, the effect of gamma irradiation at the attained efficient dose (200 Gy) was studied for three successive experiments to ensure the enhanced bioactivity constancy of the irradiated strain.

### Reaction mechanism determination by SEM

Bacterium *R. solanacearum* was washed with physiological buffer solution (PBS) and fixed with 3.5% glutaraldehyde. After that, the fixed *R. solanacearum* was washed repeatedly with PBS and rinsed with ethanol at 27 °C for 20 min before dehydration. Finally, *R. solanacearum* was fixed and set over the aluminum stump to start the SEM imaging. The treated and untreated *R. solanacearum* morphological and surface features with the biofabricated SiO_2_/Ag nanocomposite was investigated using SEM imaging.

### Statistical analysis

The employed statistical design of BB was constructed and analyzed by Minitab version 18 software (free trial version; https://www.minitab.com/en-us/products/minitab/free-trial/). While, results of gamma irradiation effect experiment were represented as the mean ± standard deviation (SD). The statistical significance was assessed by the one way ANOVA followed by Duncan’s test for comparing of means at 0.05 level of significance using SPSS version 22 software (IBM Corp, Armonk, New York, United States).

## Results

### Characterization of nano-SiO_2_, and SiO_2_/Ag nanocomposites

**Fig. 1:**
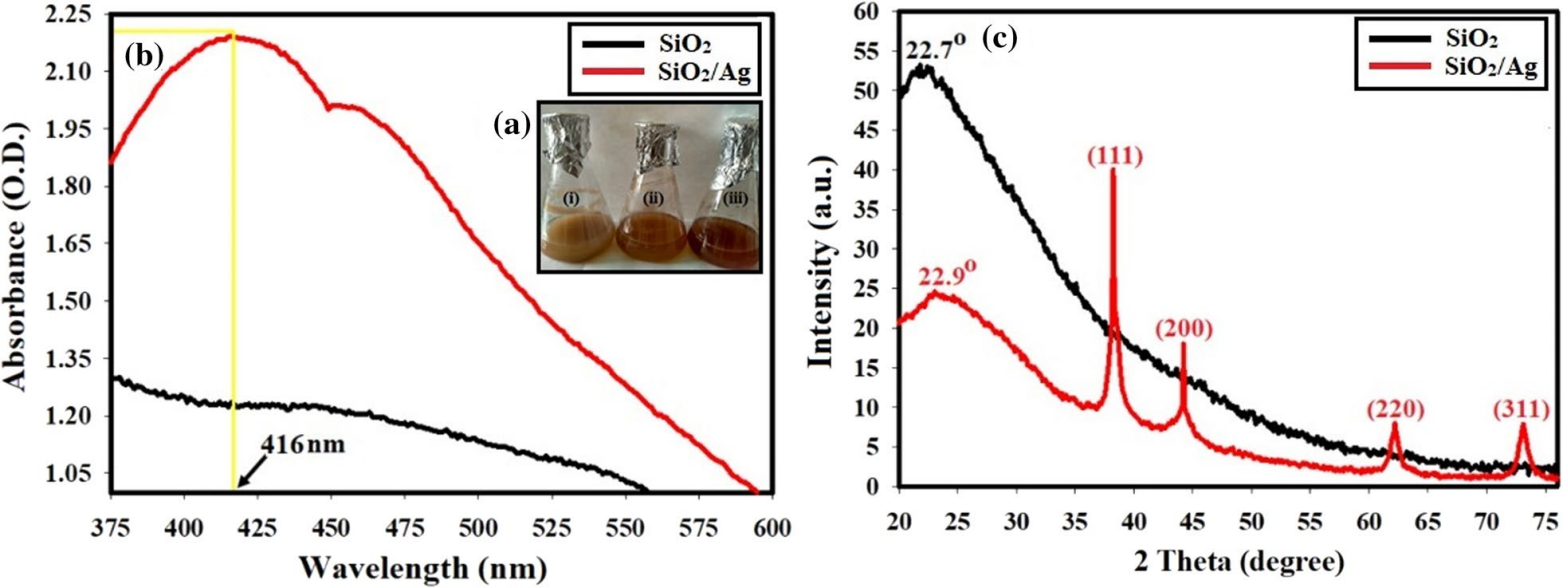
**a** The observed change in the reaction color from (i) faint brown color (non-fermented rice husk) to (ii) brown color (*F. oxysporum*-fermented rice husk and nano-SiO_2_ spheres biosynthesis) to (iii) reddish brown color (*F. oxysporum*-fermented rice husk after addition of AgNO_3_ and formation of SiO_2_/Ag nanocomposite. **b** UV–Vis. absorption spectra of SiO_2_, and SiO_2_/Ag nanocomposite showing maximum absorbance peak at 416 nm. **c** XRD patterns of the bioleached SiO_2_ and the biofabricated Ag/SiO_2_ composites, under SSF by *F. oxysporum*

The silica bioleaching process from rice husk under SSF by *F. oxysporum* for 24 h resulted in color change from a faint brown (non-fermented rice husk) to a brown color suspension (*F. oxysporum*-fermented rice husk and nano-SiO_2_ spheres biosynthesis) which then changed to a reddish brown color after reducing the silver ions in AgNO_3_ solution on nano-SiO_2_ spheres to form SiO_2_/Ag nanocomposite (Fig. 1a).

UV–Vis analysis revealed that the prepared nano-SiO_2_ spheres do not show any UV–Vis. absorption in the range of 300 nm to 800 nm, but the biofabricated SiO_2_/Ag nanocomposites show an obvious absorption peak at around 416 nm (Fig. 1b).

XRD patterns of the synthesized nano-SiO_2_ spheres and SiO_2_/Ag composites are illustrated in Fig. 1c. For nano-SiO_2_ spheres, there is only a broad scattering maximum centered at 22.7°, corresponding to amorphous silica. For the prepared SiO_2_/Ag nanocomposite, besides the amorphous silica characteristic diffraction peak, it also exhibited four well-resolved diffraction peaks at 2θ angles of 38.24°, 44.17°, 63.9° and 74.19° in the range of approximately 20° to 80°, corresponding to the crystalline nature of the nano-Ag.

**Fig. 2:**
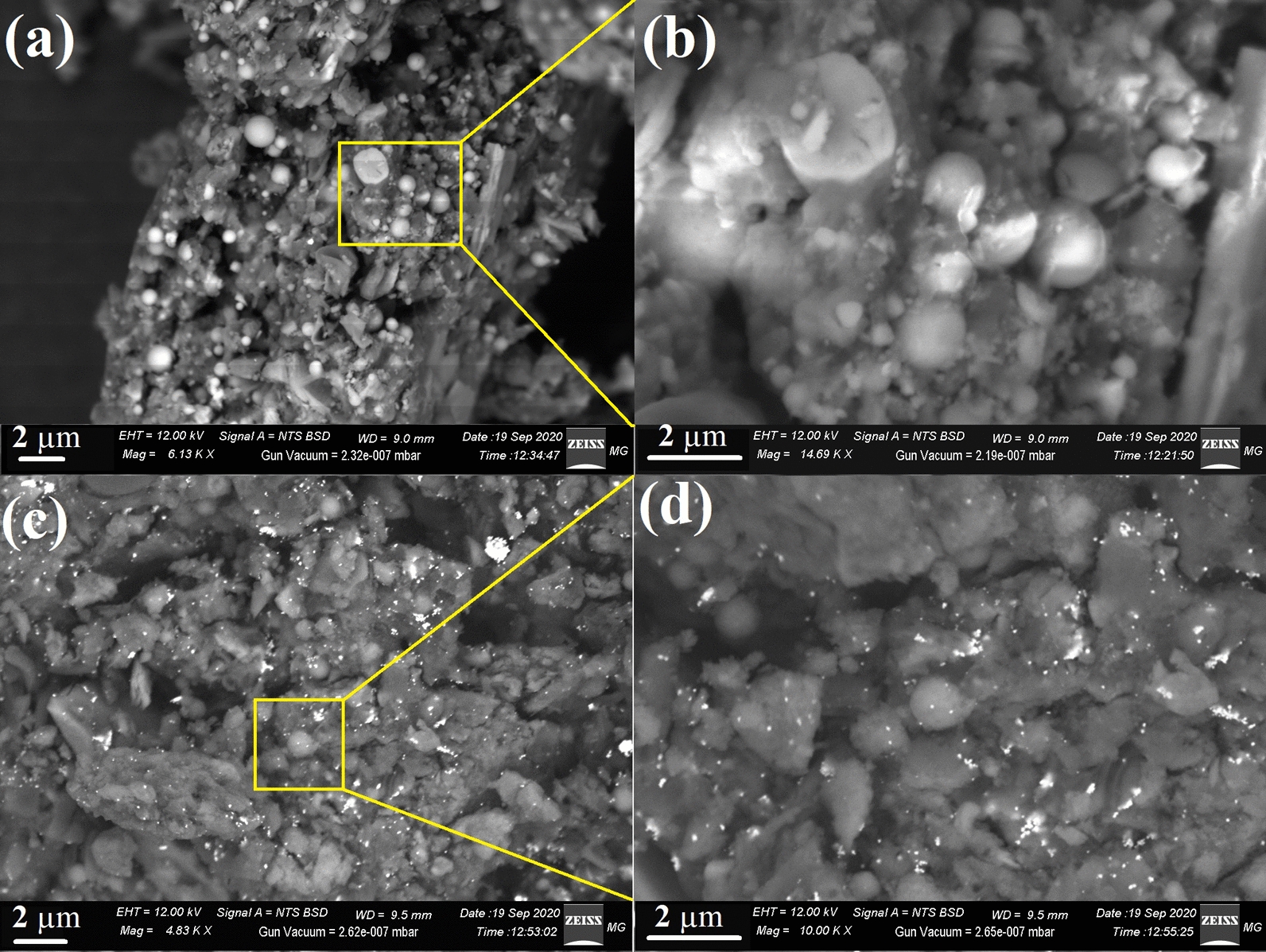
Surface morphology determination where **a** SEM imaging of the bioleached SiO_2_ **b**, the magnified area of nano-SiO_2_ sphere, **c** SEM imaging of SiO_2_/Ag nanocomposite, and **d** the magnified area of SiO_2_/Ag nanocomposite

SEM images of the prepared nano-silica show particle spheres with smooth surface and semi-homogeneous size as presented in Fig. 2a. All of the particles are spherical in shape and with different grain nano-sizes which incorporated with the fungal media as shown in Fig. 2b. For the SiO_2_/Ag nanocomposites inhabited in Fig. 2c, the silica spheres were immobilized by nano-Ag particles across the prepared nano-silica sphere. Furthermore, the dispersed composite particles in SEM remained spherical in shape with a bright particles for the loaded nano-Ag particles as appears in Fig. 2d.

**Fig. 3:**
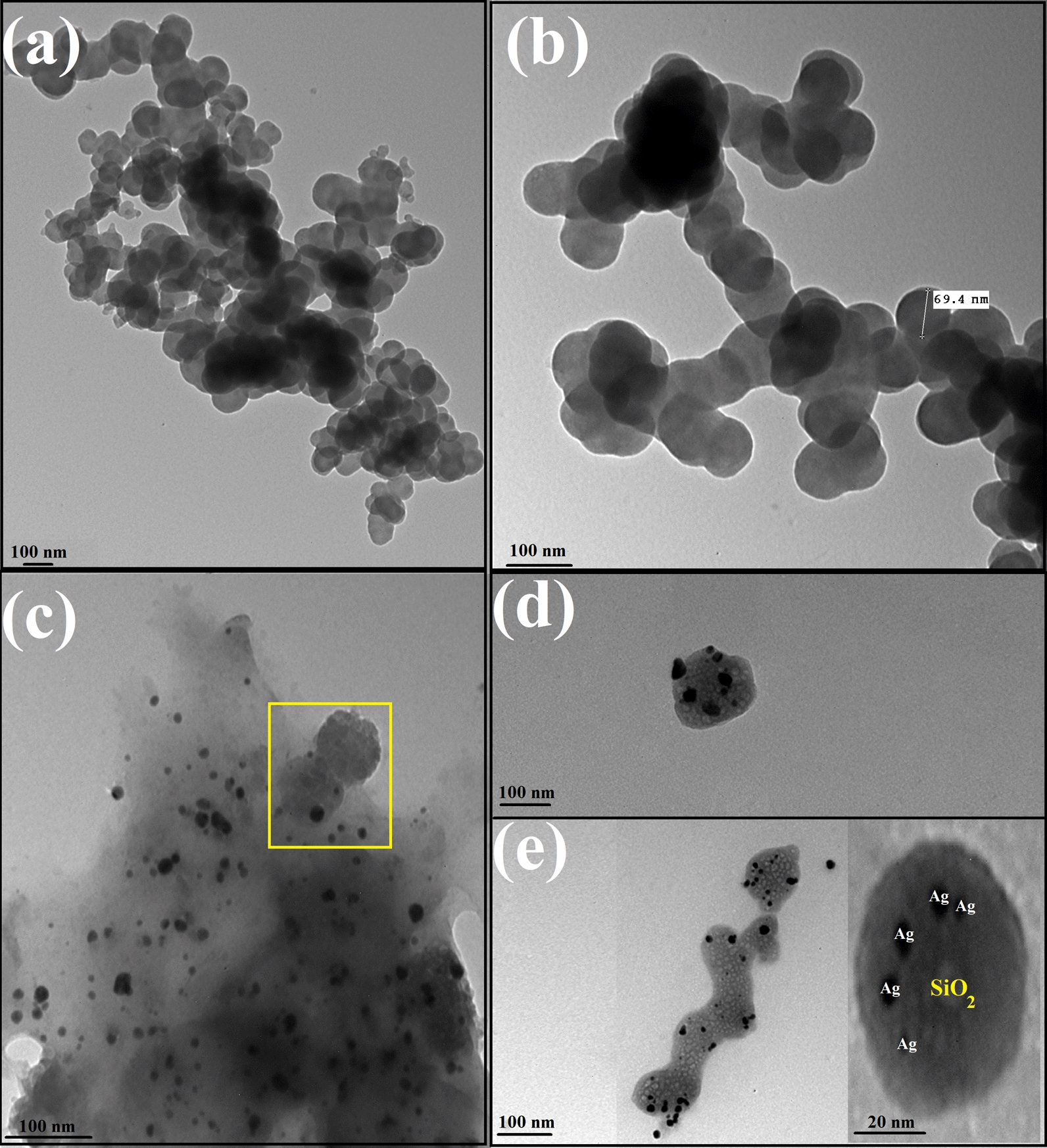
Shape, and average particle size determination where **a** and **b** TEM imaging of the bioleached SiO_2_ at different magnification position, and (**c**, **d**, and **e**), TEM imaging of SiO_2_/Ag nanocomposite at different magnification area

TEM image of the biosynthesized nano-SiO_2_, and SiO_2_/Ag nanocomposite is exhibited in Fig. 3. For the synthesized nano-SiO_2_, they own a spherical structure (Fig. 3a), with diameter sizes ranging from 93.58 nm to 42.56 nm with an average size of 69.4 nm as shown in Fig. 3b. In case of the biofabricated SiO_2_/Ag nanocomposite, the nano-Ag particles appear as black small circle particle, loaded in the faint amorphous nano-SiO_2_ spheres as presented in Fig. 3c. The magnified TEM imaging (Fig. 3d, and e), shows the successful loading of nano-Ag particles (with diameter sizes ranging from 49.85 nm to 12.95 nm with an average particle size of 25.85 nm) on the surface of nano-SiO_2_ sphere.

DLS analysis was used to evaluate particle size distribution and to calculate the average particle size of the synthesized nano-SiO_2_, and SiO_2_/Ag nanocomposite that was found as 72.11 nm, and 78.56 nm, respectively as shown in Additional file 1: Fig. S1 a, and b, respectively. It is important to state that, the grown moderate mono-size distributed nano-SiO_2_ and SiO_2_/Ag nanocomposite were attributed to the synthesis method, and the particle size distribution was slightly-increased as nano-Ag particles loaded in the prepared sample. The polydispersity index (PDI) can be obtained from instruments that use DLS or determined from electron micrographs. In the obtained PDI values (Additional file 1: Fig. S1), we found that the PDI value increased as nano-Ag particles content increased in the prepared sample, and was found to be 0.412 in nano-SiO_2_, and slightly-increased to be 0.444 in the synthesized SiO_2_/Ag nanocomposite. The present values (0.412, and 0.444) indicated that the biosynthesized samples (nano-SiO_2_ and SiO_2_/Ag nanocomposite) were moderate mono-size distributed.

**Fig. 4:**
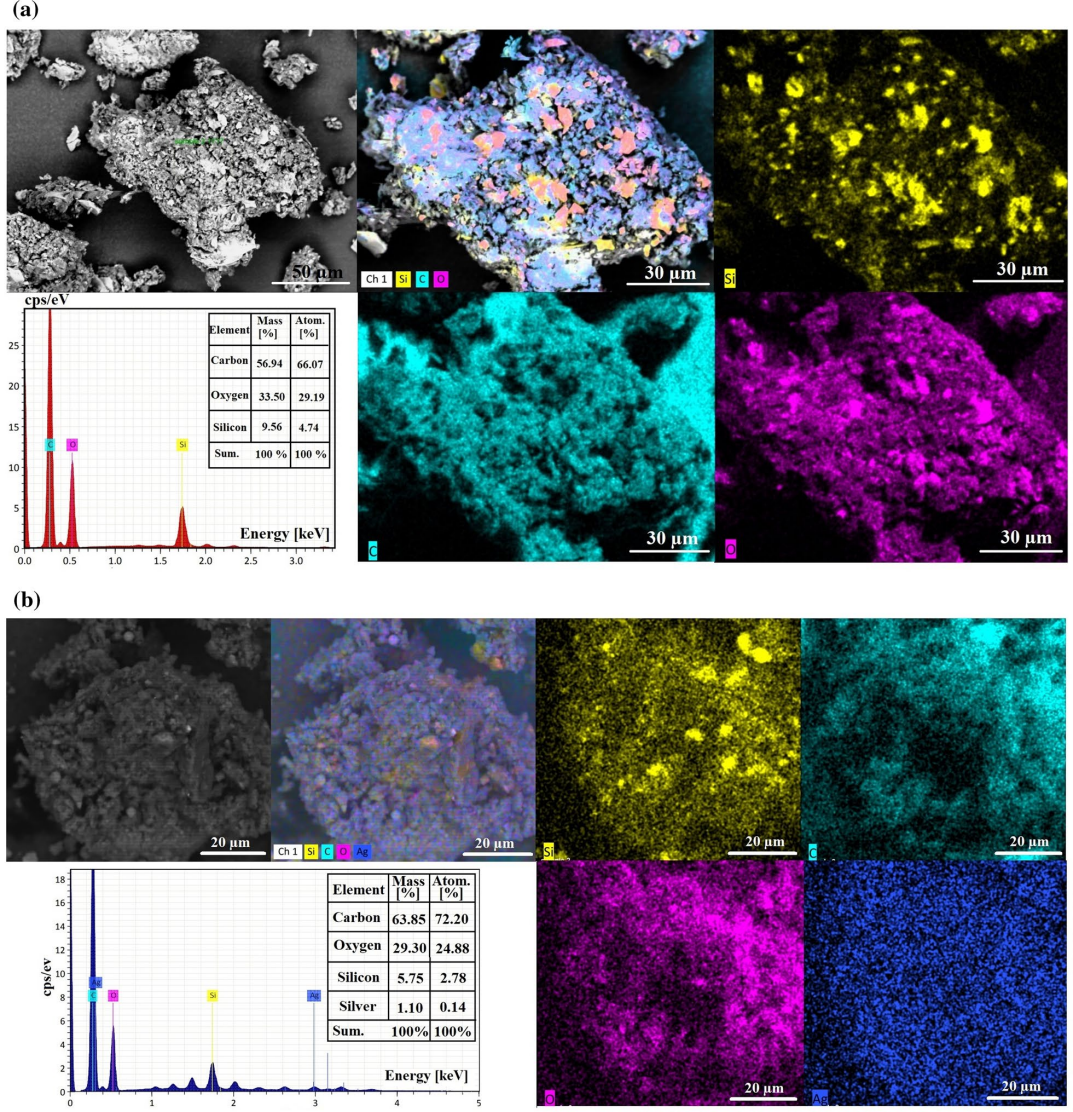
Purity, elemental analysis and mapping distribution analysis where **a** SEM/EDX spectrum of the bioleached SiO_2_, and **b** SEM/EDX spectrum of SiO_2_/Ag nanocomposite

The chemical composition, and elemental mapping of nano-SiO_2_ and SiO_2_/Ag nanocomposite has been analyzed by SEM/EDX elemental analysis as shown in Fig. 4. In this pattern, C, O, and, Si peaks were clearly shown in the EDX elemental analysis of nano-SiO_2_, and elemental mapping distribution confirmed the purity and equal distribution of the biosynthesized nano-SiO_2_ (Si, and O atoms for nano-SiO_2_, and C, and O for the remaining fungal media) as shown in Fig. 4a. On the other hand, C, O, Si, and Ag peaks were clearly shown in the elemental analysis of SiO_2_/Ag nanocomposite, and elemental mapping distribution confirms the purity, equal distribution, and successful loading of nano-Ag particles (blue color) on the synthesized nano-SiO_2_ spheres (Si, and O atoms for nano-SiO_2_, Ag atom for nano-Ag particles, and C, and O for the remaining fungal media) as shown in Fig. 4b. The atomic ratio of Si and O was about 1: 8.9, and the total content of Ag element was about 1.10 mass % (Fig. 4b).

**Fig. 5:**
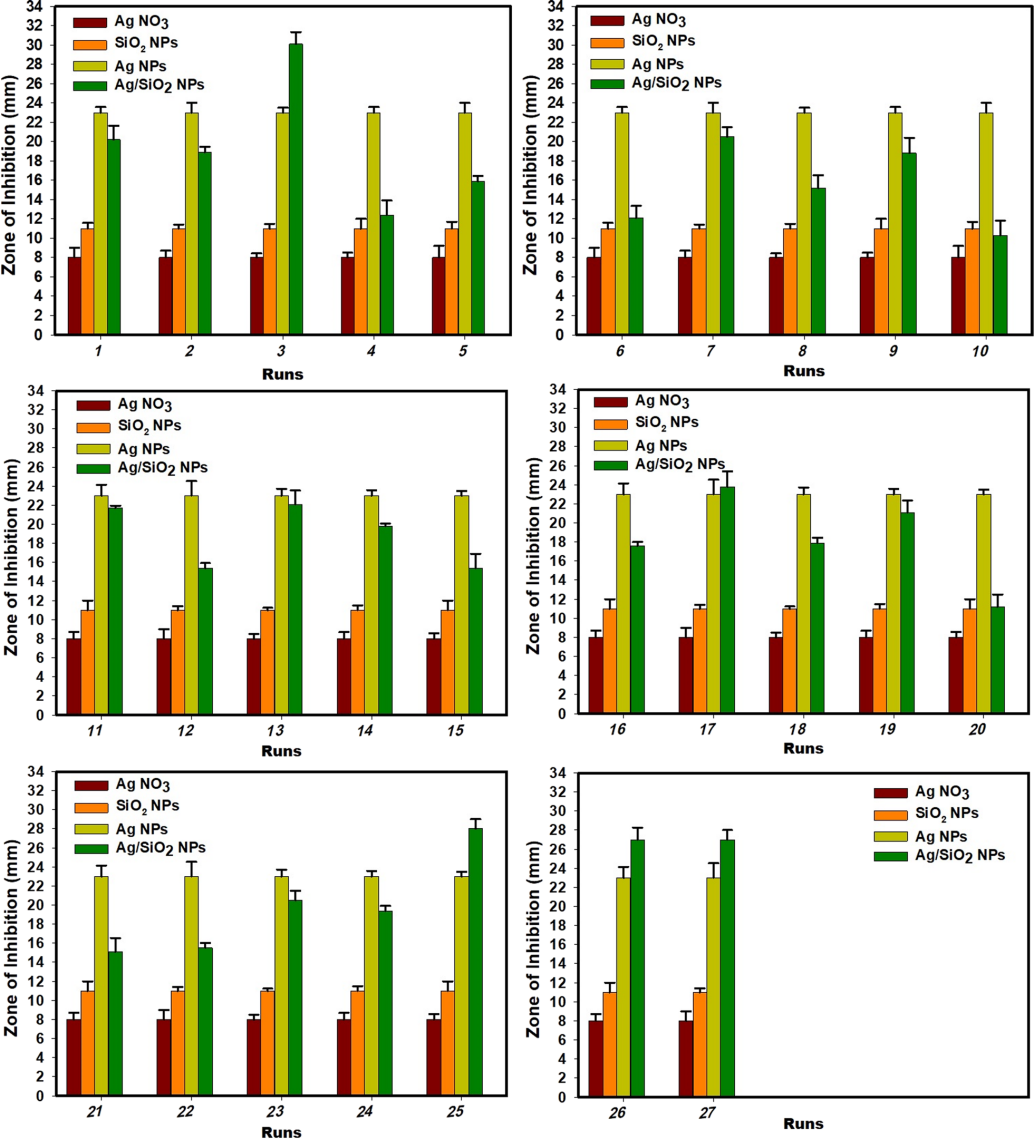
Histograms illustrated the obtained Zone of inhibition (ZoI, mm) values of the synthesized Ag/SiO_2_ nanocomposite at each experimental trial with respect to controls

### Box-Behnken design

The conditions applied for optimizing SiO_2_/Ag nanocomposite biofabrication along with experimental (actual) and predicted values for anti-*R. solanacearum* activity represented by ZoI measures in mm were recorded in Table 1. Moreover, the recorded ZoI values at each experimental run along with the ZoI values of AgNO_3_ solution, free nano-Ag, and nano-SiO_2_ were graphically-plotted in Fig. 5. Analysis of variance (ANOVA) employing Fisher's statistical analysis was used to assess the model's significance and adequacy, and the reached results are shown in Table 2. The model F value attained was 81.92 with *P* value < 0.05 indicating that it is significant. Moreover, values obtained for *P* value (< 0.05) showed that the model terms A, C, D, A^2^, B^2^, C^2^, D^2^, AB, BC, and AC were significant and were graphically represented in a pareto chart in Supplemental Fig. S2 a. Higher R^2^ value (0.9896), adjusted R^2^ value (0.9776), and predicted R^2^ value (0.9437) verify a high and adequate precision of the model and ensure that the proposed model closely matched the experimental data. Additionally, the *P* value of lack-of-fit (0.386; > 0.05) was non-significant which reveals goodness of fit. The polynomial equation created by the employed model for the relation between the studied factor and the response was as following:

**Table 2:** Analysis of Variance (ANOVA) for the estimated response (zone of inhibition)

Source	DF	Adj SS	Adj MS	*F*-Value	*P*-Value
Model	14	684.488	48.892	81.92	0.000
Linear	4	320.223	80.056	134.14	0.000
A: RH Conc.	1	205.013	205.013	343.52	0.000
B: AgNO_3_ Conc.	1	0.563	0.563	0.94	0.350
C: pH	1	62.563	62.563	104.83	0.000
D: Reaction time	1	52.083	52.083	87.27	0.000
Square	4	285.627	71.407	119.65	0.000
A2	1	80.947	80.947	135.63	0.000
B2	1	56.189	56.189	94.15	0.000
C2	1	135.789	135.789	227.53	0.000
D2	1	230.271	230.271	385.84	0.000
2-way interaction	6	78.638	13.106	21.96	0.000
AB	1	67.240	67.240	112.67	0.000
AC	1	4.000	4.000	6.70	0.024
AD	1	1.210	1.210	2.03	0.180
BC	1	5.063	5.063	8.48	0.013
BD	1	0.562	0.562	0.94	0.351
CD	1	0.563	0.563	0.94	0.351
Error	12	7.162	0.597		
Lack-of-Fit	10	6.495	0.649	1.95	0.386
Pure Error	2	0.667	0.333		
Total	26	691.650			

*DF* degree of freedom, *adj SS* adjusted sum of squares, *adj MS* adjusted mean of squares

The analysis of variance (ANOVA) was applied at 95% confidence intervals, variables and models would be statistically considerable at levels of significance, *P*-value < 0.05

Lack-of-Fit value is non-significant

$${\varvec{Z}}{\varvec{o}}{\varvec{I}}\boldsymbol{ }\left({\varvec{m}}{\varvec{m}}\right)=\boldsymbol{ }-120.6\boldsymbol{ }+4.577\boldsymbol{ }{\varvec{A}}+17.27\boldsymbol{ }{\varvec{B}}+27.96\boldsymbol{ }{\varvec{C}}+29.64\boldsymbol{ }{\varvec{D}}-\boldsymbol{ }0.1558\boldsymbol{ }{{\varvec{A}}}^{2}-3.246\boldsymbol{ }{{\varvec{B}}}^{2}-2.243\boldsymbol{ }{{\varvec{C}}}^{2}-6.571\boldsymbol{ }{{\varvec{D}}}^{2}-0.8200\boldsymbol{ }{\varvec{A}}{\varvec{B}}-0.1333\boldsymbol{ }{\varvec{A}}{\varvec{C}}+0.1100\boldsymbol{ }{\varvec{A}}{\varvec{D}}+0.750\boldsymbol{ }{\varvec{B}}{\varvec{C}}-0.375\boldsymbol{ }{\varvec{B}}{\varvec{D}}-0.250\boldsymbol{ }{\varvec{C}}{\varvec{D}}$$

**Fig. 6:**
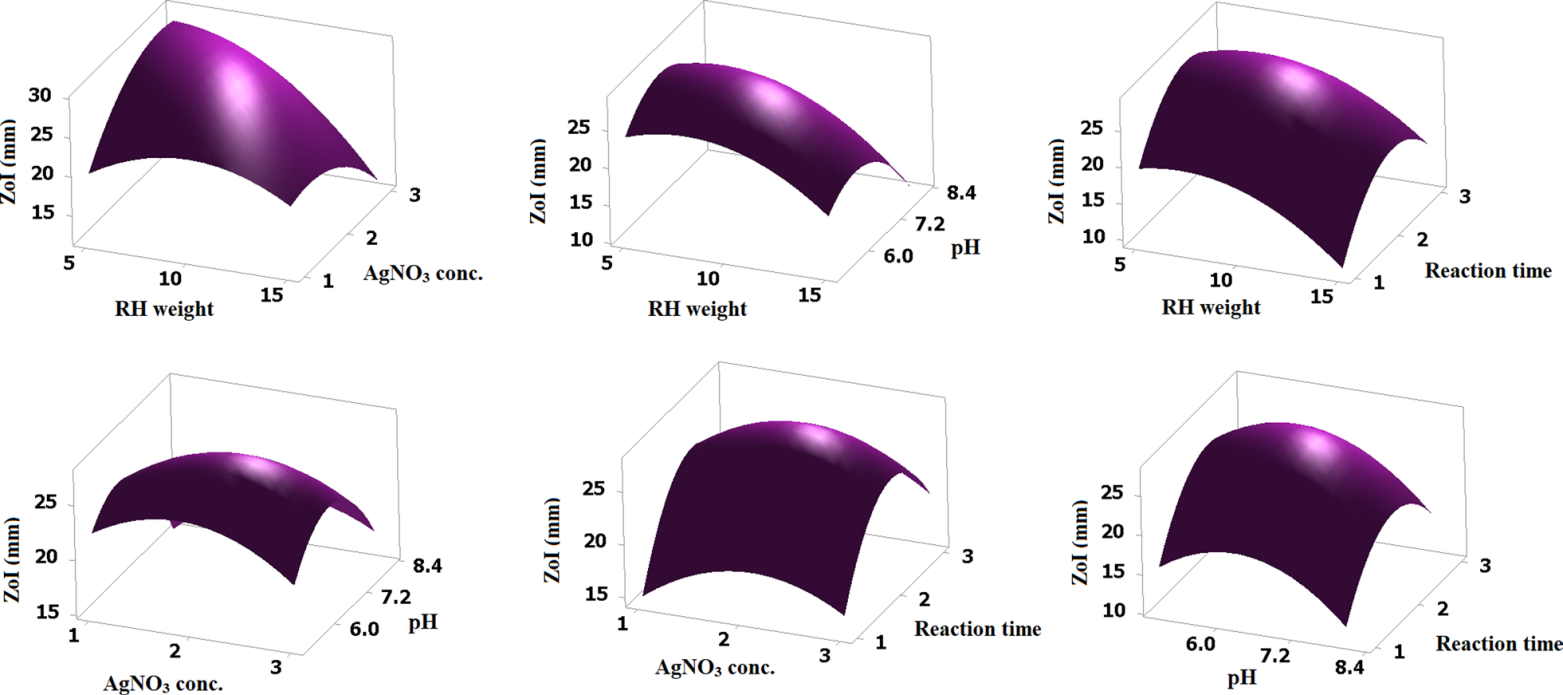
3D surface plots for visual observation of the interacted effects between levels of each two factors while the third factor was kept at the center level

A solution of the employed parameters (RH concentration, 6 g/250 mL flask; AgNO_3_ conc., 2.50 mM; reaction pH, 6.3; reaction time 2 days) with maximum desirability of 95.07% was selected by the response optimizer tool of Minitab software as presented in Supplemental Fig. S2 b and the experiments were run for validation. The obtained ZoI value, 29.50 mm was close by 98.71% to the predicted ZoI value, 29.12 mm. Figure 6 shows the 3D surface plots that correspond to the interactions between the different process parameters and their effect on the antibacterial activity of the biofabricated SiO_2_/Ag nanocomposite. ZoI in mm was plotted on the Z-axis against any two independent factors while the other three variables were set at their relevant central points to generate a response surface. As a result of considering all conceivable combinations, six response surfaces were designed (Fig. 6). Generally, the plots demonstrates that the interaction between associated variables was significant and helpful for optimizing the biofabrication process of SiO_2_/Ag nanocomposite with maximum anti-*R. solanacearum* activity.

### Gamma irradiation effect in improving the bioactivity of *F. oxysporum*

The results shown in Table 3 illustrate the influence of exposing the used *F. oxysporum* strain to gamma-radiation at various dosages, 200, 400, 600, and 800 Gy, separately, in improving its activity in the biofabrication process under SSF and as a result enhancing the anti-*R. solanacearum* activity of the biofabricated SiO_2_/Ag  nanocomposite. The gamma irradiation effect was shown to be dose-related, according to the findings. Furthermore, 200 Gy was the optimal irradiation dose for obtaining significant variations in the anti-bacterial activity of the biofabricated SiO_2_/Ag nanocomposite. Since, at this dose, the recorded ZoI (33.6 ± 0.57^a^ mm) was higher significantly (*P* < 0.05) compared to that attained by the non-irrradiated culture (28.6 ± 0.57^b^ mm). Moreover, the findings revealed the increasing of the irradiation dose from 400 to 800 Gy resulted in a significant (*P* < 0.05) decline in the bactericidal activity of the biofabricated SiO_2_/Ag nanocomposite. By repeating the irradiation experiment at the effective irradiation dose (200 Gy) compared to the control cultures, data showed that there is a significant *(P* < 0.05) stability in the irradiation effect in enhancing the bioactivity of the tested fungal strain (Table 3). Thus, results ensured that the efficiency of the biosynthesized SiO_2_/Ag nanocomposite by the irradiated strain against *R. solanacearum* was significantly higher than that obtained by control strain (non-irradiated) across the three tested trials.

**Table 3:** The effect of gamma irradiation at different tested doses on the anti-*Ralstonia solanacearum* activity by the biofabricated SiO_2_/Ag nanocomposite

Gamma irradiation dose (Gy)	ZoI (mm) 1st trial	ZoI (mm) 2nd trial	ZoI (mm) 3rd trial
0	28.6 ± 0.57^b^	30.4 ± 0.50^b^	28.5 ± 1.00^b^
200	33.6 ± 0.57^a^	34.6 ± 0.40^a^	34.1 ± 1.25^a^
400	24.0 ± 1.0^c^	ND	ND
600	19.6 ± 0.57^d^	ND	ND
800	14.0 ± 1.0^e^	ND	ND

*ND* not detected

Data for ZoI are shown as the mean ± SD of triplicate measurements from independent experiments

a–e means with different superscripts in the same column are considered statistically different (*P* ≤ 0.05)

### SEM antibacterial reaction mechanism

**Fig. 7:**
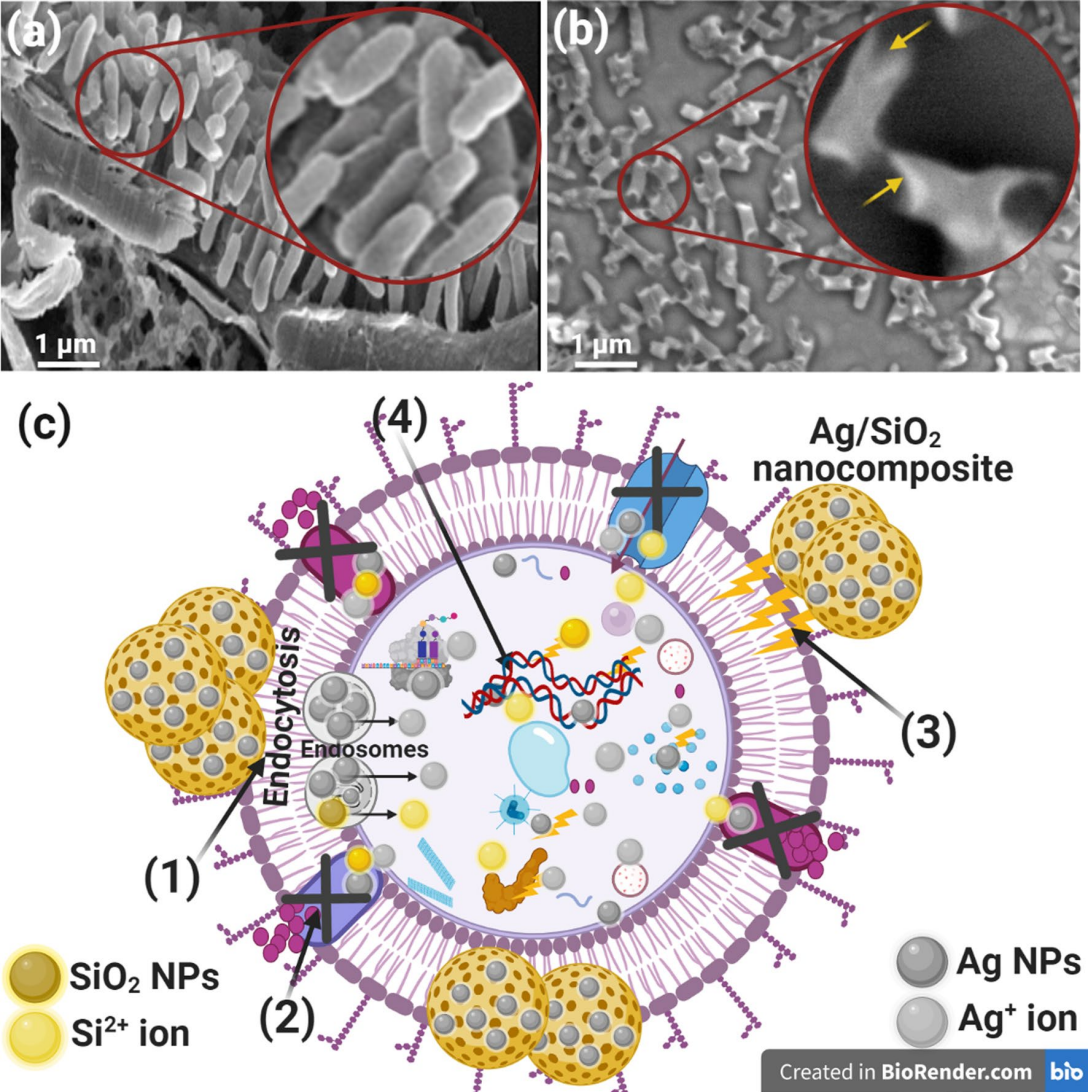
Reaction mechanism determination using SEM analysis where **a** control untreated *R. solanacearum*, **b** treated *R. solanacearum* with the biosynthesized Ag/SiO_2_ nanocomposite, and **c** schematic indication about the four significant forms of the antibacterial possibility of Ag/SiO_2_ nanocomposite, where: **1. **Ag/SiO_2_ nanocomposite attaches to the *R. solanacearum* surface and results in membrane injury and limited transport movement. 2. Ag/SiO_2_ nanocomposite stop the ions transportation to and from the *R. solanacearum*. 3. Ag/SiO_2_ nanocomposite develop and improve the ROS directing to *R. solanacearum* wall crack. 4. Silver nanoparticles (Ag NPs), and silica nanoparticles (SiO_2_ NPs) liberated from Ag/SiO_2_ nanocomposite penetrate inside the *R. solanacearum* and interact with cellular organelles, thereby influencing individual cellular machinery and modulating the cellular signal design and inducing cell death. Ag/SiO_2_ nanocomposite may serve as a vehicle to effectively deliver Ag^+^, and Si^+2^ ions to the bacterial cytoplasm and membrane, where proton motive force would reduce the pH to be less than 3.2 and enhance the liberation of ions.

SEM analysis was directed to indicate the potential antibacterial mechanism against *R. solanacearum*, as noted in Fig. 7. The SEM study regarding the control bacterial cells in the absence of biofabricated SiO_2_/Ag nanocomposite presented bacterial groups typically prolonged and grown with a standard shape and count with the whole regular surface, as displayed in Fig. 7 a.

After SiO_2_/Ag nanocomposite treatment, unusual morphological irregularities are identified in *R. solanacearum* (Fig. 7b), including the semi-lysis of the outer surface in some bacterial cells established by deformations of the *R. solanacearum* cells. On the other hand, the biofabricated SiO_2_/Ag nanocomposite performed the complete lysis of the bacterial cell and cell malformation, decreasing the total viable number (Fig. 7b) and creating holes on the surface of bacterial cells, and white layers are formed over the bacterial cells due to the chemisorption attractions between the active SiO_2_/Ag nanocomposite and the bacterial cell wall.

## Discussion

Nano-Ag particles have been found to be highly antibacterial agents. The intricate interaction of nano-Ag particles and released Ag^+^ with bacterial cells explains why Ag has a lesser chance to generate bacterial resistance. The interaction of nano-Ag particles with bacterial cell membranes results in creation of “pits” and damage to the membranes, which increases membrane permeability and causes bacterial death. Furthermore, nano-Ag particles can produce free radicals, which damage membranes and have an antimicrobial impact. Ag^+^ ions can also bind with phosphorus moieties in DNA, preventing bacterial reproduction, as well as thiol groups in bacterial enzymes and sulfur-containing proteins in bacterial cell walls, causing damage and deactivation (Sondi and Salopek-Sondi 2004; Kim et al. 2007b; Tang and Zheng 2018). However, the antibacterial activity of nano-Ag particles is generally limited by surface oxidation and aggregation, which reduces their practical uses. Nano-Ag particles tend to agglomerate at small diameters, < 20 nm, due to their large surface-to-volume ratio and high surface energy, which reduces their antibacterial activity. This difficulty has been addressed by researching silver-carriers such as titanium dioxide, zeolite, activated carbon, and silica, and these efforts have greatly increased their antimicrobial effectiveness (Abdel Maksoud et al. 2021, 2022; Bekhit et al. 2022; Qasim et al. 2015).

Silica particles have been shown to be an effective stabilizing matrix for obstructing nano-Ag particles accumulation. Furthermore, SiO_2_ particles have great chemical and thermal stability, as well as being inert and biocompatible, making them an effective delivery vehicle, in the form of silica nanospheres and nanotubes; silica shell; and silica thin film, for antibacterial agents (Akhavan and Ghaderi 2009; Camporotondia et al. 2013). In addition, a significant number of silanol (Si–OH) groups on the surface of SiO_2_ have hydrophilic properties, allowing for strong hydrogen bonding interactions with any polar substance (Nur Kamilah et al. 2019). In this study, we produced a composite by immobilizing nano-Ag particles on mesoporous nano-SiO_2_ particles to enhance the advantages of immobilization and prolong the release of Ag NPs to preserve the antibacterial properties in agricultural applications.

Nano-Ag particles on bulk matrices show a reduced washing resistance resulting in rapid release of Ag^+^ ion in a short time process of their antibacterial action. To avoid nano-Ag particles aggregation and acquire a long-standing antibacterial activity, nano-Ag particles could be loaded in the pores of porous material (Zhang et al. 2004; Akhavan and Ghaderi 2009) or coated by a core–shell structure as silicon (Alimunnisa et al. 2017). The use of core–shell structures is one method for nano-Ag immobilization. The aggregation of Ag cores when SiO_2_ shell thickness is reduced and the slow dissolving rate of Ag cores when shell thickness is increased are the key problems in this strategy (Ung et al. 1998). Such properties may limit Ag NPs from being fully employed in core–shell systems. On the other hand, Ag^+^ ions have been demonstrated to exhibit sustained release from immobilized nano-Ag particles on substrates, indicating that immobilization of nano-Ag particles can give long-term antibacterial effects. The prolonged Ag release from the SiO_2_/Ag nanocomposite and leaching profile of Ag from SiO_2_/Ag nanocomposite was studied by Mosselhy et al. (2017) using an inductively coupled plasma-optical emission spectrometer (ICP-OES). The total concentration of Ag in 1 mg (SiO_2_/Ag composite)/mL was 57.8 ± 10.4 µg Ag/mL. A concentration of 7.5 ± 1.2 µg Ag/mL (13% of the stock Ag concentration) was detected at the start of the experiment (0 h). Ag was rapidly leaked from the composite after 24 h, with a concentration of 22.1 ± 2.3 µg Ag/mL (38.2%). After that, a slower persistent leaching of Ag was discovered, with concentrations of 27.1 ± 2.4 µg Ag/mL (46.9%) after 48 h and 28.4 ± 2.2 µg Ag/mL (49.1%) after 72 h. The depletion of immobilized nano-Ag particles from the surface could be a possible elucidation for the subsequent delayed sustained release of Ag.

UV–Vis. spectroscopy is one of the most widely used techniques for structural characterization of Ag nanostructures (El-Batal et al. 2020a). The absorption peak obtained at 416 nm of the currently prepared nanocomposite was due to the Mie plasmon resonance excitation from the nano-Ag particles on the surface of SiO_2_ (Cortijo-Campos et al. 2020). Compared to pure Ag NPs, the absorption peak shifted from ~ 400 nm to 416 nm (Baraka et al. 2017; El-Batal et al. 2018). The possible reason for the red shift can be attributed to much larger size of Ag NPs and higher coverage on the silica surface (Wang et al. 2013; Liu et al. 2020). The UV–Vis. results are consistent with previous reports (Amendola et al. 2010; Wang et al. 2013; Ansari et al. 2019; Liu et al. 2020). The strong dipole–dipole interactions between neighboring NPs and Mie scattering of silver shell would promote red shift and broadening of the plasmon bands for Ag clusters attached on silica spheres (Tang et al. 2007; Ashurov et al. 2019). XRD patterns ensured the fabrication of SiO_2_/Ag nanocomposites with the an amorphous silica broad scattering peak at 22.7° (Wu et al. 2016; Sakthisabarimoorthi et al. 2017) besides four diffraction peaks which can be indexed to the (111), (200), (220), and (311) reflections of face-centered cubic metal silver, and corresponding to joint committee on powder diffraction standards (JCPDS) card no. 04–0783 (Adur et al. 2018), indicating that the nano-Ag particles with high crystallinity could be obtained on the surface of SiO_2_. In addition, the peaks were a little broader than that of bulk silver because the silver size is relatively small (El-Batal et al. 2019). From the SEM results we can conclude that the morphological surface shape of nano-SiO_2_ and nano-Ag in the biosynthesized SiO_2_/Ag nanocomposites does not change as the prepared nano-SiO_2_ spheres and the grain size were presented to be in nano-scale and this corresponds with the formerly published articles (Oh et al. 2006; Wang et al. 2011; Mourad et al. 2020). Additionally, TEM analysis revealed that our biosynthesized SiO_2_/Ag nanocomposites own the approximate same size, and spherical shape as those in the recently published article which confirmed that the shape and size are applicable for many biological applications (Kim et al. 2005, 2007a; Oh et al. 2006; Otari et al. 2016). DLS size measurements were higher than particle size measurements in TEM. DLS analysis is estimating the hydrodynamic radius of nano-SiO_2_ and SiO_2_/Ag nanocomposite bounded by water molecules, resulting in larger particle sizes of the capped nanoparticles, while TEM is calculating the average particle size of the powder material without the water layer (Abdel Maksoud et al. 2020). Based on the obtained PDI values, the prepared particles in the nanocomposite showed moderate mono-size dispersion. The international standards organizations (ISOs) have established that PDI values < 0.05 are more common to monodisperse samples, while values > 0.7 are common to a broad size (e.g., poly-disperse) distribution of particles (Franks et al. 2019). SEM–EDX elemental mapping also confirmed the preparation of nanocomposite as reported in recent studies (Kim et al. 2005; Alimunnisa et al. 2017).

According to literature, SiO_2_/Ag composites were fabricated via different chemical and physical methods. The fabrication process based on primarily synthesizing of SiO_2_ NPs from chemical silica precursors as tetraethyl orthosilicate (TEOS) mostly through a sol–gel technique-based Stötber method. Then, nano-Ag was synthesized through chemical, physical or biological reduction of silver ions on the surface of the prepared nano-SiO_2_ spheres either via a one-step process or they were synthesized separately and then added to the prepared nano-SiO_2_ spheres with continuous stirring for enhancing the immobilization of nano-Ag on the SiO_2_ nano-spheres resulting in fabrication of SiO_2_/Ag nanocomposite. Accordingly, Kim et al. (2005) applied the Stötber method for synthesizing Ag–SiO_2_ NPs with nano-SiO_2_ particles of 200 nm and nano-Ag particles of less than 10 nm. Akhavan and Ghaderi (2009) synthesized Ag/SiO_2_ thin films via a sol–gel protocol with average particles size of 58 nm. Oh et al. (2006) applied gamma-irradiation to reduce Ag^+^ in AgNO_3_ solution and synthesize nano-Ag on the surface of nano-SiO_2_ obtained by sol–gel protocol. Qin et al. (2017) synthesized nano-SiO_2_ particles by a modified sol–gel method then applied a chemical reduction process of AgNO_3_ solution using sodium citrate as reducing agent to obtain nano-Ag on nano-SiO_2_ spheres. Tang et al. (2007) fabricated SiO_2_/Ag core–shell composite particles via a simple and one-step ultrasonic electrodeposition protochol.

Towards biological fabrication processes, a one-step synthesis of nano-Ag particles with SiO_2_ core shell (Ag@SiO_2_) has been developed in an environmentally friendly manner (Otari et al. 2016). A fermented grain mash product was employed as a silver ion reducing agent and a catalyst for the creation of SiO_2_ shells on nano-Ag particles (Otari et al. 2016). In addition, synthesis of nano-SiO_2_ spheres from biomass as rice husk can provide a low-cost alternative to TEOS-based synthesis process (Prabha et al. 2019). In addition, Cui et al. (2016) reported of a facile creation of rice husk-based nano-SiO_2_ particles, synthesized via a hydrothermal method, and coated with nano-Ag synthesized by chemical reduction. Bioleaching of silica from rice husk and extracellular synthesis of nano-SiO_2_ spheres using collected biomass of cultivated *F. oxysporum* under submerged fermentation was reported (Bansal et al. 2006). Accordingly, the current study is the first one to develop a whole green method for SiO_2_/Ag nanocomposite biofabrication based on *F. oxysporum*-fermented rice husk under SSF.

Statistical optimization approaches like response surface methodology (RSM) are commonly used to investigate responses after simultaneous variation of multiple process parameters (Tortella et al. 2019; Zaki et al. 2019; Saeed et al. 2020; Zaki and El-Sayed 2021). By employing the response surface methodology, the nanocomposite fabrication showed a better response to variations in the different investigated parameters as pH, reaction time, AgNO_3_ concentration and RH concentration. Here, the response surface plot provided a simple and convenient tool to illustrate the main and interaction effect of the significant parameters on the employed biofabrication process as well as define the optimum levels for proficient nanocomposite synthesis under SSF with a maximum anti-bacterial activity.

Previous reports synthesized Ag NPs with anti-bacterial activity towards *R. solanacearum* to control bacterial wilt (Chen et al. 2016; Tortella et al. 2019; Cheng et al. 2020). The SiO_2_/Ag nanocomposite or Ag@SiO_2_ core shell synthesized in previous studies displayed a wide range of antimicrobial activity towards different human and phyto-pathogen. Quang et al. (2011) prepared nano-Ag particles-containing SiO_2_ micro beads with a good antibacterial effect towards *Escherichia coli.* Qasim et al. (2015) reported anti-candidal activity against *Candida albicans* of nano-Ag particles-embedded mesoporous nano-SiO_2_ spheres. Otari et al. (2016) demonstrated the antibacterial activity of core shell Ag@SiO_2_ nanoparticles against *Staphylococcus aureus*, *Bacillus cereus*, and *E. coli.* Additionally, an anti-*Xanthomonas oryzae* pv. *oryzae* (a plant pathogen that causes bacterial leaf blight of rice) activity by SiO_2_/Ag nanocomposite was also reported (Cui et al. 2016).

Gamma rays can cause mutations in cell genes, resulting in an overexpressing of the secondary metabolites (Unluturk 2016) responsible for silica bioleaching as well as Ag salt reduction. Here, 200 Gy gamma radiation dose was shown to be optimal for increasing anti-bacterial activity of the biofabricated nanocomposite by irradiated *F. oxysporum-*fermented rice husk, with significant variations in the obtained ZoI values. From literature, a gamma irradiation dose of 1000 Gy was applied to *Monascus purpureus* resulting in improving the biosynthesis yield of selenium NPs under SSF (El-Sayed et al. 2020b) as well as enhancing the biosynthesis of cobalt-ferrite nanoparticles using cell free filtrates from irradiated culture under submerged fermentation (El-Sayed et al. 2020a). Previous studies proved that the gamma irradiation at appropriate dosages can improve the production of a variety of fungal metabolites (El-Sayed et al. 2020c; Zaki et al. 2021).

The schematic diagram in Fig. 7c shows the possible antibacterial mechanism, and had been conducted by the Biorender program (https://biorender.com). There were important and excellent potential actions such as reactive oxygen species (ROS) production due to the Ag loaded in the synthesized SiO_2_/Ag nanocomposite (El-Batal et al. 2018). It is recommended that the synthesized Ag/SiO_2_ nanocomposite adhere to the bacterial cells by chemisorption, and ROS will be developed, which could change the bacterial cell morphology, reduce the bacterial membrane permeability and provide the occupancy of oxidative stress genes about their replies because of the ROS production (Abdel Maksoud et al. 2020). We realize that SiO_2_/Ag nanocomposites begun their movement by bonding at the surface of the *R. solanacearum*, permitting membrane damage, construction of pits, and switching off the ions transportation activity (El-Sayyad et al. 2020). Then, the formation of ROS inside the *R. solanacearum* moved to the corresponding ions in the microbial cell, damaging all intracellular structures like DNA, plasmid, and various critical bacterial organelles. Then cellular toxicity happened because of the oxidative stress created by ROS production (Abd Elkodous et al. 2021).

Although there are many previous studies on the synthesis of SiO_2_/Ag nanocomposites, this study benefited for the first time from the dual role of the fermented rice husk (FRH). Since, FRH is a non-expensive substrate for nanosilica synthesis as well as it plays an important role in reducing AgNO_3_ to Ag NPs to be immobilized on nano-silica spheres. This one-pot nanocomposite biofabrication method is novel and has not been reported in the literature. The study also benefited from the advantages of solid state fermentation (SSF) which is rarely reported in the NPs synthesis. In SSF, the microorganism cultivated under low moisture content that drive the microorganism to produce active compounds and extracellular enzymes that play an important role in NPs synthesis. Also, through SSF, the whole culture (biomass and fungal metabolites) is used in the NPs synthesis to achieve an efficient biosynthesis process.

In conclusion, this study revealed an alternative method for biofabrication of SiO_2_/Ag nanocomposite through solid state fermentation (SSF) of rice husk by *F. oxysporum* that has not yet been reported. This SSF-dependent approach is green and non-expensive as it frequently uses solid wastes from agro-industrial systems as the substrate. In this biofabricated SiO_2_/Ag nanocomposite, nano-Ag particles are embedded in mesoporous nano-SiO_2_ spheres to avoid Ag NPs aggregation and delay their release. Obtained results from UV–visible spectra, DLS, XRD, SEM–EDX, and TEM showed that the surface of nano-SiO_2_ spheres was efficiently immobilized by nano-Ag particles. Response surface methodology was effective in detecting the optimum levels responsible for proficient nanocomposite bio-fabrication and maximum anti-*R. solanacearum* activity. To the best of our knowledge, application of a statistical model to optimize the biofabrication of SiO_2_/Ag nanocomposite for maximum bioactivity has not been reported anywhere. Furthermore, gamma irradiated *F. oxysporum* at a dose of 200 Gy showed more enhanced bioactivity than that of the non-irradiated strain.

Future work will be directed to recover mutant strains of *F. oxysporum* after irradiation at 200 Gy (efficient dose) and screen their efficiency and bioactivity stability. In addition, PCR analysis and additional molecular studies would be performed to ensure the mutation incidence and detect the mutation region in the selected mutant strain. Moreover, a field experiment will be applied to study the in vivo protective and therapeutic effect of the biofabricated nanocomposite on plants susceptible to the *Ralstonia* wilt.

## Supplementary Information

**Additional file 1:** **Figure S1.** Particle size distribution, hydrodynamic radius, and polydispersity index (PDI) determination where (a) DLS analysis of the bioleached SiO_2_, and (b), DLS analysis of SiO2/Ag nanocomposite. **Figure S2.** (a) Is the Pareto Chart of the standardized single, squared, and interacted effects of the studied variables. A: RH Conc.; B: AgNO_3_ Conc.; C: pH; D: Reaction time, and (b) is the response surface optimizer for predicting the optimum levels of the studied variables for generating maximum response (antibacterial activity) by the biofabricated SiO_2_/Ag nanocomposite.

## Data Availability

The datasets used and analyzed during the current study are available from the corresponding author on reasonable request.
