# Supplementary material for: Novel fabrication of SiO2/Ag nanocomposite by gamma irradiated Fusarium oxysporum to combat Ralstonia solanacearum
Source: AMB Express. 2022 Feb 28;12:25. doi: 10.1186/s13568-022-01372-3 (PMC8885861; doi:10.1186/s13568-022-01372-3)
Supplement: Supplementary file 1 — Additional file 1: Figure S1. Particle size distribution, hydrodynamic radius, and polydispersity index (PDI) determination where (a) DLS analysis of the bioleached SiO2, and (b), DLS analysis of SiO2/Ag nanocomposite. Figure S2. (a) Is the Pareto Chart of the standardized single, squared, and interacted effects of the studied variables. A: RH Conc.; B: AgNO3 Conc.; C: pH; D: Reaction time, and (b) is the response surface optimizer for predicting the optimum levels of the studied variables for generating maximum response (antibacterial activity) by the biofabricated SiO2/Ag nanocomposite. [file 13568_2022_1372_MOESM1_ESM.pdf]

AMB Express

Novel fabrication of SiO_2/Ag nanocomposite by gamma irradiated *Fusarium oxysporum* to combat *Ralstonia solanacearum*

Amira G. Zaki[1], Yasmeen A. Hasanien[1], Gharieb S. El-Sayyad[2*]

[1]Plant Research Department, Nuclear Research Center (NRC), Egyptian Atomic Energy Authority (EAEA), Cairo, Egypt.

[2]Drug Radiation Research Department, National Center for Radiation Research and Technology (NCRRT), Egyptian Atomic Energy Authority (EAEA), Cairo, Egypt.

*Corresponding author: Gharieb S. El-Sayyad, Email: Gharieb.S.Elsayyad@eaea.org.eg; ORCID: 0000-0001-5410-7936; Tel: +201022938593

Supplementary figures:

Fig. S1: Particle size distribution, hydrodynamic radius, and polydispersity index (PDI) determination where (a) DLS analysis of the bioleached SiO_2, and (b), DLS analysis of SiO_2/Ag nanocomposite**.**

Fig. S2: (a) is the Pareto Chart of the standardized single, squared, and interacted effects of the studied variables. A: RH conc.; B: $AgNO_3$ conc.; C: pH; D: Reaction time, and (b) is the response surface optimizer for predicting the optimum levels of the studied variables for generating maximum response (antibacterial activity) by the bio fabricated SiO_2/Ag nanocomposite.

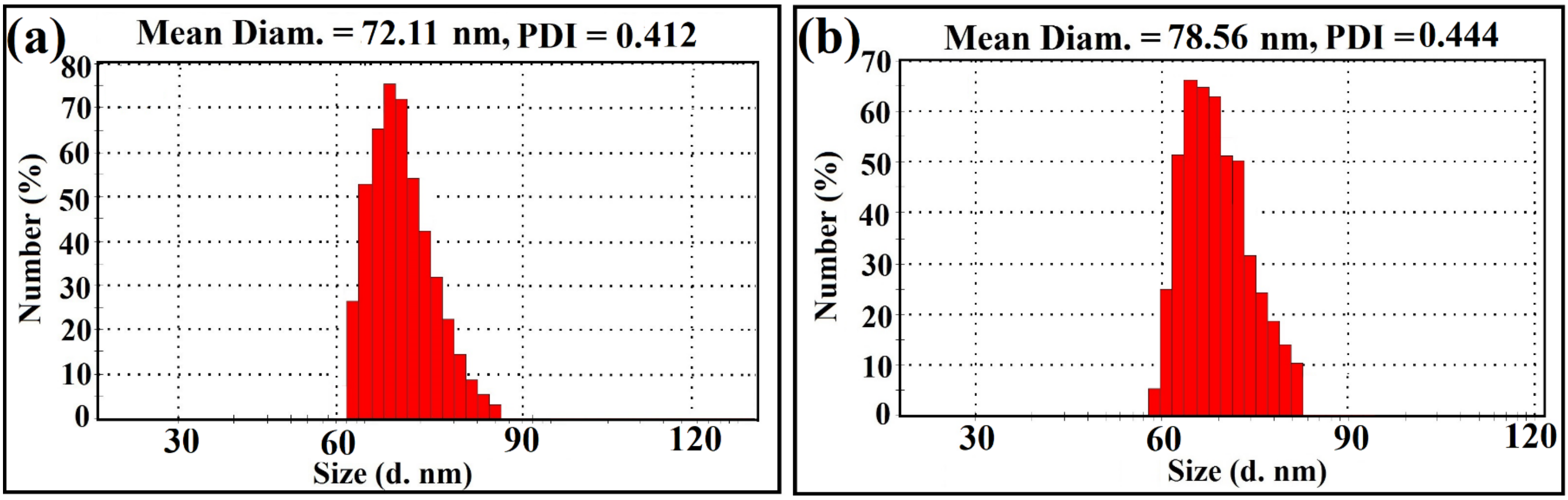

Fig. S1: Particle size distribution, hydrodynamic radius, and polydispersity index (PDI) determination where (a) DLS analysis of the bioleached SiO_2, and (b), DLS analysis of SiO_2/Ag nanocomposite**.**

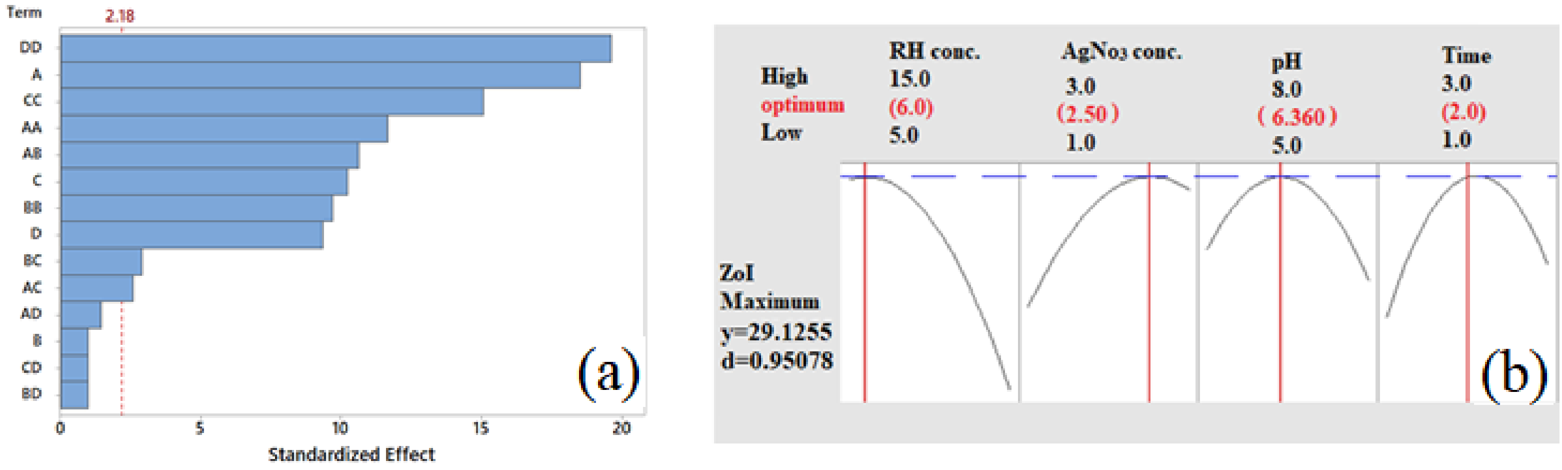

Fig. S2: (a) is the Pareto Chart of the standardized single, squared, and interacted effects of the studied variables. A: RH conc.; B: $AgNO_3$ conc.; C: pH; D: Reaction time, and (b) is the response surface optimizer for predicting the optimum levels of the studied variables for generating maximum response (antibacterial activity) by the bio fabricated SiO_2/Ag nanocomposite.
